# Relationship between mental health and climacteric adjustment in middle aged women: a confirmatory analysis

**DOI:** 10.1186/s12905-023-02397-x

**Published:** 2023-05-06

**Authors:** Maedeh Khakkar, Ashraf Kazemi

**Affiliations:** 1 Student Research Committee, School of Nursing and Midwifery, Isfahan University of Medical Sciences, Isfahan, Iran; 2 Reproductive Health Departments, Nursing and Midwifery Care Research Center, School of Nursing and Midwifery, School of Nursing and Midwifery, Isfahan University of Medical Sciences, Isfahan, Iran

**Keywords:** Climacteric, Adjustment, Mental health, Middle aged

## Abstract

**Background:**

Climacteric changes in women are associated with an increased probability of psychological symptoms. Identifying the relationship between adjustment to this period and mental health helps to plan for middle-aged women’s health improvement. Therefore, the present study aimed to investigate the relationship between climacteric adjustment (CA) and mental health in middle aged women.

**Method:**

This cross-sectional study was conducted on 190 women aged 40 to 53 years. Mental health symptoms (including hypochondriasis, anxiety, depression, and social impairment) and CA were assessed using 28-item general health questionnaire and the CA questionnaire, respectively, as a self-report. Data were analyzed using linear and stepwise regression methods, and the fitting of the resulting conceptual model was assessed using AMOS software.

**Results:**

The results showed that hypochondriasis score and social impairment, anxiety level and CA in the perfection dimension, and social impairment score and CA in perfection, decline in beauty, and sexual silence dimensions had an inverse relationship. Moreover, the relationship between anxiety score and CA in the reaction to end of menstruation and the relationship between social impairment and decline of femininity were positive and significant. Factor analysis of the conceptual model obtained from the study results demonstrated a good model fit (CMIN /DF = 0.807, P = .671).

**Conclusion:**

The results showed a relationship between CA and psychological symptoms in middle-aged women. In other words, the level of hypochondriasis, anxiety, and social impairment symptoms decreased with increasing CA in sexual silence, perfection, and decline in beauty.

## Abstract (summary)

Climacteric changes in women are associated with increased psychological symptoms. Identifying the relationship between adjustment to this period and mental health helps to plan for middle-aged women’s health improvement. Therefore, the present study aimed to investigate the relationship between climacteric adjustment (CA) and mental health in middle aged women. This cross-sectional study was conducted on 190 women aged 40 to 53 years. Mental health symptoms (including hypochondriasis, anxiety, depression, and social impairment) and CA were assessed using 28-item general health questionnaire and the CA questionnaire, respectively, as a self-report. The results showed that hypochondriasis score and social impairment, anxiety level and CA in the perfection dimension, and social impairment score and CA in perfection, decline in beauty, and sexual silence dimensions had an inverse relationship. Moreover, the relationship between anxiety score and CA in the reaction to end of menstruation and the relationship between social impairment and decline of femininity were positive and significant. The results showed a relationship between CA and psychological symptoms in middle-aged. In other words, the level of hypochondriasis, anxiety, and social impairment symptoms decreased with increasing CA in sexual silence, perfection, and decline in beauty.

## Background

The climacteric period in middle-aged women is the menopausal transition period associated with significant physiological and psychological changes [1]. In this period, reproductive changes such as decreased fecundity and approaching menopause, and physical changes in the sexual organs following the decline of sex hormones, affect the middle-aged women’s body image [2] and reduce their sexual satisfaction [3, 4]. The feeling of femininity decline and the need to repair the relationship with the husband was one of the experiences that middle-aged women expressed following changes in their fertility [5].

Fertility and sexual attractiveness are essential criteria for playing feminine gender roles, whose decline in middle-aged women can reduce marital satisfaction [6] and cause a higher prevalence of psychological disorders in middle-aged women than men [7]. In addition, following the decreased ovarian activity and menopause, the risk of cardiovascular diseases [8] and decreased sleep quality [9] enhances, which may be associated with psychological complications such as depression.

The relationship between middle-aged women’s fertility changes and their mental health [10] shows that improving their mental health requires more accurate identification of the relationship between the factors affecting this cycle. Gender roles may influence psychological reactions related to menopause, such as depression and stress [11], and climacteric changes affecting these roles may convert menopause into a critical period. The results of meta-analysis studies showed that the pooled prevalence of depression in menopausal Chinese [12] and Indian women were 36.3% and 42.47% Indian women [13], respectively.

Therefore, adjustment to changes in fertility in middle-aged women may help alleviate this crisis. Adjustment is an efficient process for managing stress in the face of a crisis and justifies individuals’ psychological responses to the crisis [14]. Accordingly, the climacteric adjustment (CA), as an adjustment to changes in fertility, may act as a mediator to modify the negative effects of coping with these changes and middle-aged women’s mental health.

Therefore, the present study was conducted to investigate the relationship between climacteric adjustment (CA) and psychological symptoms in middle-aged women. The research questions were whether the climacteric adjustment and its dimensions are related to psychological symptoms in middle-aged women.

## Materials and methods

### Design and participants

This cross-sectional study was conducted on middle-aged women aged 40 to 53 years, referred to Health Centers in Isfahan, Iran, between June 2021 and November 2021 to receive women health care. Inclusion criteria included no life crises based on Holmes criterion and no major psychological illnesses (such as schizophrenia, delusional or substance-induced psychotic disorder) based on the health record. The sample size was estimated as 190 participants with a test power of 85% and a reliability of 95%. Also, according to a pilot study, a correlation coefficient between adjustment and mental health was estimated to be 0.3.

### Sampling

Sampling was performed in two stages: selecting health centers and sampling middle-aged women. A total of 10 centers were selected from among the health centers of Isfahan through a stratified random sampling method. Between 18 and 20 eligible women, from each center, were randomly selected from middle-aged women receiving health care in these centers. The women were selected based on their record numbers using a table of random numbers. After selecting the participants, the purpose of the study was explained via telephone, and if they met the inclusion criteria, they were invited to participate in the research. On the appointment, informed consent was obtained from them, and after completing the demographic questionnaire, their CA and mental health were assessed using a questionnaire.

### Instruments

Mental health was assessed using general health questionnaire-28 (GHQ-28). This valid questionnaire includes seven questions for each psychological symptoms including depression, anxiety, social impairment, and hypochondriasis dimensions [15], with the internal consistency of 91% for Iranian population [16].

Adjustment to climacteric was measured using the valid CA questionnaire with 6 dimensions indicating the reaction to end of menstruation, perfection, decline in beauty, lack of sexual attraction, decline in femininity, and sexual silence. This questionnaire consists of 34 items on a 5-point Likert scale, including 5 = strongly agree, 4 = agree, 3 = neutral, 2 = disagree, and 1 = strongly disagree. In this questionnaire, a higher score indicates higher adaptation. A previous study reported an internal reliability of 0.863 for the questionnaire [17].

### Statistical analysis

Statistical analysis was performed using windows-based SPSS software version 19. The statistical methods included linear and stepwise regression. The conceptual model was developed based on the relationship between mental health and the dimensions of CA. Moreover, the fit of the model was calculated using the AMOS version 19.

## Results

Of the 198 middle-aged women invited, 190 women aged between 40 and 53 accepted participating in the study. Most of the study subjects had higher education, and 25.8% experienced menopause. The results of the descriptive data are presented in Table 1. Evaluating the relationship between participants’ contextual variables and psychological symptoms using stepwise regression revealed that the total score of psychological symptoms, hypochondriasis, anxiety, and depression, had a positive and significant relationship with hot flashes. The spouse’s age and education, menopause, dyspareunia, number of children, and the woman’s employment were excluded from the model. Multivariate linear regression showed that regardless of hot flashes, the score of psychological symptoms was inversely related to the perfection and decline in beauty dimensions (Table 2).

**Table 1:** Descriptive results of data analysis

Variables	Mean (SD) Or Number (%)
Age (year)	46.3 (4.7)
Education (%)	
Less than high school	16 (8.4)
High school	63 (33.2)
University degree	111 (58.4)
Occupied (%)	94 (49.4)
Menopause (%)	49 (25.8)
Hot flash (%)	79 (41.6)
Psychological health	29.9 (8.5)
Hypochondriasis	7.8 (3.2)
Anxiety	7.3 (3.6)
Depression	3.3 (2.1)
Social impairment	11.5 (2.8)
Reaction to end of Menstruation	21.8 (6.8)
Perfection	15.0 (4.4)
Decline in beauty	14.3 (4.7)
Lack of sexual attraction	7.9 (3.7)
Decline of femininity	5.8 (2.4)
Sexual silence	12.6 (4.5)

**Table 2:** Association between total score of psychologic health and climacteric adjustment dimensions

	Psychologic symptom (total score)			
	R^2^_Adj_ = 0.19; F = 7.53; P < .0001			
	Beta	Sig	Confidence Interval 95.0%	
			Lower	Upper
Reaction to end of menstruation	0.14	ns	− 0.002	0.42
Perfection	− 0.28	< 0.0001	-1.03	− 0.32
Decline in beauty	− 0.14	0.04	− 0.59	− 0.01
Lack of sexual attraction	− 0.06	ns	− 0.67	0.24
Decline of femininity	− 0.02	ns	− 0.73	0.53
Sexual silence	− 0.14	ns	− 0.66	0.01
Hot flash	0.26	< 0.0001	2.84	8.45

The results showed that the hypochondriasis score was inversely dependent on adaptation in the sexual silence dimension. In addition, the anxiety score had a positive and significant relationship with CA in the reaction to end of menstruation dimension and a negative relationship with perfection. Social impairment had an inverse relationship with Perfection, decline in beauty, and sexual silence; however, its relationship with adaptation in the decline of femininity dimension was positive and significant. The results showed that, regardless of climacteric adjustment dimensions, the relationship between hot flashes and hypochondriasis, anxiety, and depression scores was positive and significant (Table 3).

**Table 3:** Association between psychologic symptoms and climacteric adjustment dimensions

	Hypochondriasis				Anxiety				Depression				Social impairment			
	R^2^_adj_ = 0.11, F = 3.66, P < .0001				R^2^_adj_ = 0.16, F = 5.46, p < .0001				R^2^_adj_ = 0.08, F = 3.21, P = .003				R^2^_adj_ = 0.07, F = 3.26, P = .005			
	Beta	Sig	95% CI		Beta	Sig	95% CI		Beta	Sig	95% CI		Beta	Sig	95% CI	
			Lower	Upper			Lower	Upper			Lower	Upper			Lower	Upper
**Hot flash**	0.28	0.001	0.93	3.28	0.22	0.005	0.62	3.43	0.16	0.02	0.15	2.17	0.07	ns	− 0.45	1.21
**Adjustment**																
Factor 1	0.01	ns	− 0.07	0.08	0.22	0.003	0.06	0.25	0.05	ns	− 0.05	0.10	0.08	ns	− 0.03	0.10
Factor 2	− 0.09	ns	− 0.21	0.06	− 0.35	< 0.0001	− 0.53	− 0.21	− 0.15	ns	− 0.25	0.01	− 0.17	0.04	− 0.22	− 0.01
Factor 3	− 0.04	ns	− 0.15	0.06	− 0.10	ns	− 0.23	0.04	− 0.10	ns	− 0.18	0.04	− 0.16	0.04	− 0.18	− 0.01
Factor 4	− 0.03	ns	− 0.22	0.13	0.01	ns	− 0.19	0.23	− 0.14	ns	− 0.32	0.01	− 0.03	ns	− 0.17	0.11
Factor 5	− 0.04	ns	− 0.31	0.18	0.06	ns	− 0.40	0.17	− 0.10	ns	− 0.37	0.09	0.18	0.02	0.03	0.42
Factor 6	− 0.18	0.04	− 0.26	− 0.01	0.03	ns	− 0.20	0.12	− 0.30	ns	− 0.15	0.10	− 0.20	0.01	− 0.24	− 0.03

**Definitions**: Factor 1: Reaction to end of menstruation; Factor 2: Perfection; Factor 3: Decline in beauty; Factor 4: Lack of sexual attraction, Factor 5: Decline of femininity; Factor 6: Sexual silence

**Abbreviations**: adj: adjusted; CI: confidence interval; Sig: significant

According to the research results, the default model fit was evaluated using AMOS software, and the results showed that reaction to end of menstruation had a correlation with decline of femininity and lack of sexual attraction. The results also showed that anxiety levels had a positive relationship with the perfection dimension of CA as well as with reaction to end of menstruation. Social impairment was positively dependent on decline of femininity; however, it was negatively dependent on decline in beauty, sexual silence, and perfection. The hypochondriasis dimension of psychologic symptoms was negatively dependent on sexual silence. Moreover, the results of data analysis showed that the indirect effect of CA dimensions on psychological symptoms was not significant (Table 4).

**Table 4:** Regression Weights: (Default model)

				Direct Effects		
			Standardized regression weights	Estimate	Critical ratio	Sig
Decline of femininity	<---	Reaction to end of menstruation	0.201	0.071	0.003	0.003
Lack of sexual attraction	<---	Reaction to end of menstruation	0.150	0.068	0.034	0.034
Social impairment	<---	Decline of femininity	0.200	0.248	0.008	0.008
Social impairment	<---	Decline in beauty	− 0.138	− 0.085	0.055	0.055
Social impairment	<---	Sexual silence	− 0.212	− 0.140	0.005	0.005
Anxiety	<---	Perfection	− 0.311	− 0.327	0.000	< 0.0001
Hypochondriasis	<---	Sexual silence	− 0.188	− 0.156	0.002	0.002
Anxiety	<---	Reaction to end of menstruation	0.199	0.133	0.001	0.001
Social impairment	<---	Perfection	− 0.141	− 0.097	0.062	0.062

**Fig. 1:**
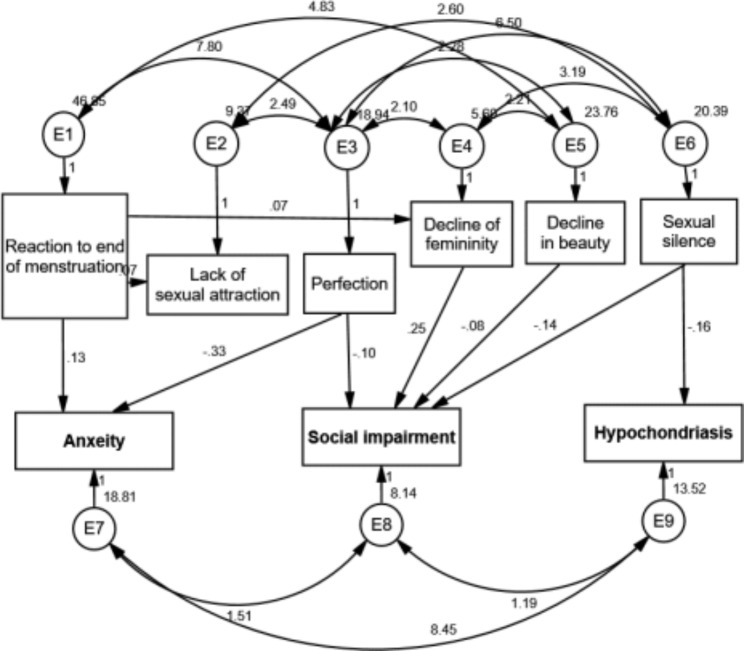
Conceptual model of relations between climateric adjustment and mental health

The overall structural equation model (Fig. 1) represented good fit for the model indicating the relationship between CA dimensions and social impairment, anxiety and hypochondriasis (CMIN = 12.11, CMIN /DF = 0.807, P = .671).

## Discussion

This study aimed to determine the relationship between psychological symptoms and climacteric adaptation in middle-aged women. The results showed that women’s psychological symptoms, including hypochondriasis, anxiety, and social impairment, were correlated with climacteric adaptation. Of the above disorders, social impairment had a more significant relationship with climacteric adaption than other disorders. These results confirm the importance of adaptation to climacteric changes for middle-aged women’s well-being [18] and show that adapting to new body conditions and sexual relationships can improve women’s mental health. Therefore, it is essential to pay attention to climacteric adaptation in mental health promotion programs for middle-aged women.

The first main finding of the study was that among the assessed psychological disorders, hypochondriasis was negatively dependent on adaptation in the sexual silence dimension. This finding shows that women who were not adapted to decreased quality of sexual activity at the climacteric experienced a more negative mentality about their physical health. Although the present study results showed no correlation between dyspareunia and hypochondriasis, the incompatibility between men’s and women’s sexual orientations during middle age and the husbands’ sexual desires may be accompanied by coercion for women and their reluctance to have a sexual relationship. Previous studies have reported a relationship between sexual abuse and somatization [19, 20]. Another study likewise reported that sexual coercion was correlated with complaints about somatic symptoms [21]. This study suggests that some middle-aged women’s physical complaints may indicate their failure to adapt to sexual conditions at the climacteric.

Another finding of this study showed that the level of anxiety in middle-aged women had a positive relationship with end of menstruation and a negative relationship with the level of adjustment in the Perfection dimension. In other words, women who adapted to the onset or imminence of menopause reported higher anxiety levels. However, women who considered this stage of their lives as a period of perfection reported less anxiety. The positive relationship between anxiety levels and CA in the reaction to end of menstruation dimension is inconsistent with the research results, which indicate that adaptation to new life events is associated with greater well-being [22]. It should be taken into account that mental health in middle age can be affected by factors other than women’s condition at the climacteric.

Furthermore, one of the critical events in this period is abnormal uterine bleeding, which can reduce middle-aged women’s quality of life and increase anxiety [23]. Although dysfunctional uterine bleeding has not been considered in the present study, adaptation to menopause is probably higher in these women, explaining the positive relationship between anxiety levels and adaptation in the reaction to end of menstruation dimension.

The dependence of anxiety levels on adaptation in the perfection dimension indicates that the level of anxiety is lower in women who, by entering the climacteric period, have transcended individual values ​​beyond social norms and do not restrict themselves to the framework of female gender norms such as physical attractiveness and sexual ability. This finding confirms Bejon’s study. He believes that if an individual’s self-image is the reflection of this image in other individuals’ minds, it can destroy their self-esteem [24] and is associated with increased anxiety [25]. However, it should be noted that self-confidence, as a personality trait, can have a synergistic effect on the relationship between adaptation and anxiety in middle-aged women in the perfection dimension.

According to another finding of the study, unlike other psychological symptoms, the level of depression was not correlated with the dimensions of climacteric adaptation. This finding may reveal a stronger effect of other depression-related factors in middle-aged women. The relationship between depression and climacteric adaptation is influenced by factors such as reduced economic and social levels and lack of social support [26]; factors that threaten middle-aged individuals more than youth.

In the present study, the social disorder was more correlated to climacteric adaptation dimensions than other psychological disorders. This study showed that social impairment was negatively dependent on adaptation in perfection, decline in beauty, and sexual silence dimensions. In addition, the relationship between social impairment and adaptation in the decline of femininity dimension was positive.

The relationship between social impairment and adaptation in perfection and decline in beauty dimensions showed that women who overlooked reduced physical attractiveness to participate in social interactions were more satisfied with their social relations.

The relationship between adaptation in the dimensions of reduction in beauty and perfection with social impairment may be explained by the relationships between sociocultural attitudes and beliefs with social acceptance and criteria for beauty. Given that women’s social roles are affected by feminine characteristics, aging and lack of adaptation to climacteric changes may decline the perception of social acceptance and competence by affecting body image (MIO) and reduce the quality of women’s contributions and social activities [27].

Moreover, the negative relationship between social impairment and adaptation in the sexual silence dimension showed that women’s less adaptation to changes in the sexual dimension was associated with social impairment. This finding indicates the importance of sexuality in middle-aged women’s social health. A study has shown that sexual dysfunction is associated with a tendency to internalize, self-discredit, and limited self-concept [28].

Although no relationship was found between dyspareunia and climacteric adaptation and psychological disorders in this study, lack of adaptation to sexual silence might be associated with negative self-concept and affect social health.

The relationship between psychological symptoms and hot flashes indicates the importance of managing this climacteric complication to improve middle-aged women’s mental health. Other studies have similarly shown that hot flashes along with anxiety affect middle-aged women’s physical symptoms by influencing their sleep quality [18]. This study also showed that assessing the level of middle-aged women’s adaptation to climacteric could be considered as a criterion for predicting their mental health.

Although the observed relationships in the dimensions of chimeric adaptation and psychological symptoms were confirmed using confirmatory factor analysis, the limitations of the present study should be taken into account while interpreting the results. The first limitation of the present study was the lack of opportunity to evaluate factors such as the quality of the marital relationship, women’s personality traits, and common menopausal disorders, namely dysfunctional uterine bleeding, which can affect middle-aged women’s mental health and be associated with climacteric adaptation. Therefore, it is recommended that these variables be considered in future studies that evaluate the relationship between middle-aged women’s mental health and climacteric adaptation. Besides, the characteristics of cross-sectional studies that restrict the interpretation of results to causal relationships were the other limitation of the present study.

## Conclusion

This study showed that the physical symptoms in middle-aged women depended on their level of adaptation to climacteric changes in the sexual silence dimension. Furthermore, the anxiety level increased in the reaction to end of menstruation dimension as the adaptation increased. social impairment was also higher in middle-aged women who had less adaptation in the perfection, decline in beauty, and sexual silence dimensions. However, women who had less adaptation to decline of femininity experienced higher social impairment. These results indicated that in middle-age women, failure- to- adapt with to climacteric changes in middle-aged women may might induce psychological symptoms. Therefore, it is mandatory that policy-makers and women’s health service providers do their best to help women improve their capability to increase their adjustment to climacteric changes.

## Data Availability

Data used in this paper are not publicly available due to the the ethical standards of the ethics committee of Isfahan University of Medical Sciences. The data are available on reasonable request from the corresponding author.
